# Fear of childbirth: validation of the Kiswahili version of Wijma delivery expectancy/experience questionnaire versions A and B in Tanzania

**DOI:** 10.1186/s12884-022-05134-8

**Published:** 2022-11-29

**Authors:** Agnes F. Massae, Margareta Larsson, Sebalda Leshabari, Columba Mbekenga, Andrea B. Pembe, Agneta S. Svanberg

**Affiliations:** 1 Department of Women’s and Children’s Health, Uppsala University, Uppsala, Sweden; 2 Department of Community Health Nursing, Muhimbili University of Health and Allied Sciences, P.O. BOX 65001, Dar Es Salaam, Tanzania; 3 Faculty of Nursing and Midwifery, Hubert Kairuki Memorial University, Dar Es Salaam, Tanzania; 4 Department of Obstetrics and Gynaecology, Muhimbili University of Health and Allied Sciences, Dar Es Salaam, Tanzania

**Keywords:** Fear of childbirth, Pregnancy, Postnatal, W-DEQ, Validity, Reliability, Tanzania

## Abstract

**Background:**

Fear of childbirth is common both before and after childbirth, often leading to complications in mother and new-born. The Wijma Delivery Expectancy/Experience Questionnaires (W-DEQ) are commonly used to measure fear of childbirth among women before (version A) and after childbirth (version B). The tools are not yet validated in the Tanzanian context. This study aimed to validate the reliability, validity, and factorial structure of their Kiswahili translations.

**Methods:**

A longitudinal study was conducted in six public health facilities in the Pwani region, Tanzania. In all, 694 pregnant and 625 postnatal women were concurrently selected and responded to W-DEQ-A and W-DEQ-B. Validation involved: translating the English questionnaires into Kiswahili; expert rating of the relevancy of the Kiswahili versions’ items; computing content validity ratio; piloting the tools; data collection; statistical analysis with reliability evaluated using Cronbach’s alpha and the intraclass correlation coefficient. Tool validity was assessed using factor analysis, convergent and discriminant validity. Exploratory factor analysis and confirmatory factor analysis were conducted on data collected using W-DEQ-A and W-DEQ-B, respectively.

**Results:**

Exploratory factor analysis revealed seven factors contributing to 50% of the total variation. Four items did not load to any factor and were deleted. The factors identified were: fear; lack of self-efficacy; lack of positive anticipation; isolation; concerns for the baby; negative emotions; lack of positive behaviour. The factors correlated differently with each other and with the total scores. Both Kiswahili versions with 33 items had good internal consistency, with Cronbach’s alphas of .83 and .85, respectively. The concerns for the baby factor showed both convergent and discriminant validity. The other six factors showed some problems with convergent validity. The final model from the confirmatory factor analysis yielded 29 items with good psychometric properties (χ^2^/df = 2.26, *p* =  < .001, RMSEA = .045, CFI = .90 and TLI = .81).

**Conclusions:**

The Kiswahili W-DEQ-A-Revised and W-DEQ-B-Revised are reliable tools and measure fear of childbirth with a multifactorial structure, encompassing seven factors with 29 items. They are recommended for measuring fear of childbirth among pregnant and postnatal Tanzanian women. Further studies are needed to address the inconsistent convergent validity in the revised versions and assess the psychometric properties of W-DEQ-A among pregnant women across gestational ages.

## Statement of significance



## Introduction

Pregnancy and giving birth are positive events in most women’s and families’ lives. However, these events are associated with negative psychological responses in some women, including severe fear of childbirth (FoB). FoB is a common and complex feeling ranging from worry to extreme anxiety and involves a perceived inability to undergo the labour process and give birth [1, 2]. FoB can be primary, occurring before childbirth, or secondary, arising after experiences of traumatic or distressing childbirth. It can be low, moderate, high, severe, or extend to tokophobia/pathological fear [3–5]. According to previous studies, the rate of FoB ranges between 5 and 30% globally [6–8], and the rates in Sub-Saharan African countries are stated to be 22.1% in Kenya [9], 24.5% in Ethiopia [10] and 22% in Malawi [11].

It is suggested that FoB is multidimensional and influenced by a woman’s concern for her own well-being and that of her baby [4], labour pain [5, 12–14], loss of control during labour and birth [12], uncertainty regarding ability to bear and birth the baby, feeling lonely [5, 15], previous birth experiences and medical interventions [5, 16, 17]. Severe FoB is reported to interfere with the process and mode of delivery in subsequent childbirth, as women may opt for an elective caesarean section [18–20]. It may also lead to increased childbirth interventions [19, 21], poor maternal-infant bonding [13, 14] and post-traumatic symptoms [13, 17].

Various tools have been used in measuring FoB, including a 10-item scale called Childbirth Fear prior to pregnancy [22], which is used mainly among young adults prior to their first pregnancy and delivery, and the Fear of Birth Scale, which has two items: worried versus calm and strong fear versus no fear [23]. Other tools include single visual analogue scales asking women how afraid they are of childbirth on a scale 1–10 [16]. The tool most frequently used is the Wijma Delivery Expectancy/Experience Questionnaires (W-DEQ) [2]. The scale was designed by Wijma in 1998, in Swedish, to evaluate women’s cognitive appraisal of the childbirth process. It has two versions for assessing women’s feelings, prenatal perceptions and expectations about FoB during pregnancy (version A) and their experiences of fear during and after childbirth (version B) [2]. In recent years, the scale has been translated into several languages, particularly in Scandinavian, Asian and some Sub-Saharan African countries. The tools have been tested for psychometric properties [24–37] and are being used to assess the prevalence, predictors, associates and effects of increased FoB. The original version had 33 items designed to measure different aspects of FoB and was postulated to be a unidimensional tool [2]. Evidence from later studies using factor analysis recommended a multidimensional tool, measuring multiple dimensions of FoB. The structure ranged from 3 to 9 factors [24–34, 37], with most studies suggesting 4 factors [25, 27–29, 33, 34]. The number of items loaded to each factor and the number of items retained with adequate psychometric validity in the final versions of the questionnaires varied across studies.

Despite its variability across regions and cultures, the W-DEQ has been shown to be a reliable and valid tool with good psychometric properties for measuring FoB both before and after childbirth [30–33, 37]. The tools have been validated in some African countries, such as Malawi [38] and Kenya [39]. However, the questionnaires have not been validated for measuring FoB in the Tanzanian context. During pregnancy, labour and the postpartum period, women need holistic care covering physical, emotional, spiritual, mental and psychological aspects. To screen psychological aspects and women’s expectations and experiences of childbirth and improve the care of childbearing women, it is crucial to have a translated and validated screening tool to assess FoB in Tanzanian women. This could lead to early identification and management of women with and at risk of FoB before and after childbirth, as well as prevention of complications associated with severe FoB. This would benefit these women’s mental health and their infants’ physical health and development. Therefore, this study was carried out to translate W-DEQ-A and W-DEQ-B from English to Kiswahili and then assess reliability and validity and explore the factorial structure of the Kiswahili versions.

## Materials and methods

### Research design and settings

A longitudinal study was carried out in six public health facilities in the Pwani region. The Pwani region is located in the east and encompasses seven districts: Kisarawe, Mkuranga, Mafia, Bagamoyo, Kibiti, Rufiji and Kibaha [40]. A district hospital and two health centres from two districts with high numbers of pregnant women seeking antenatal care were randomly selected. The Kisarawe district has 40 health facilities: one district hospital, three governmental health centres and 36 dispensaries (32 governmental and 4 privately owned) [41]. The Mkuranga district has 57 health facilities: one hospital, six health centres (two governmental and four privately owned) and 50 dispensaries (37 governmental and 13 privately owned). During the data collection period, district hospitals were providing both vaginal and caesarean section services, while health centres were offering services to women giving birth vaginally. Data were collected at reproductive and child health clinics in the selected health facilities.

### Study population and sampling

The study population consisted of all pregnant women seeking antenatal care at the selected health facilities between September 2018 and July 2019. Every woman was assessed for eligibility. The inclusion criteria were: at 32 or more gestational weeks, planning to attend the same health facility for antenatal, childbirth and postnatal services, living in one of the selected districts, expecting a vaginal delivery, and being a Kiswahili speaker. Women with a previous caesarean section were excluded from the study. Eligible women were consecutively recruited until the calculated sample size was attained. The sample size was calculated by using two proportion formulae in Epi Info 7 StatCal with a power of 80% and a significance level of 0.05 (two-tailed). A minimum sample of 616 women were expected to be enrolled in this study. Of 702 eligible pregnant women, 694 agreed to participate and were interviewed, and they were followed up from pregnancy through delivery to the postnatal time. In all, 69 were lost to follow-up, with 625 completing the study and being interviewed again at 4–6 weeks post-childbirth. The loss to follow-up was due to poor communication (some women did not have mobile phones and others were unreachable), travel after birth or geographical hindrances. Others withdrew from the study due to, e.g., not getting permission from their male partners to continue with the study or infant death.

### Instruments

The Kiswahili translations W-DEQ-A and W-DEQ-B were used in data collection. A questionnaire with sociodemographic (age, education, occupation, income and marital status) and obstetric questions (gravidity, parity, pregnancy status and complications of previous pregnancy) was also administered. W-DEQ-A and W-DEQ-B are self-report measurements of FoB before and after childbirth, respectively [2]. Each questionnaire consists of 33 items rated on a 6-point Likert scale ranging from 0 (not at all) to 5 (extremely). The minimum total score is 0, and the maximum is 165. The higher the score, the more intense the FoB. The tool developer proposed cut-off points as follows: a score of ≤ 37 reflects a low level of fear, a score of 38–65 reflects a moderate level of fear, a score of 66–84 reflects a high level of fear, and a score of ≥ 85 indicates a severe level of fear [1].

The W-DEQ-A and W-DEQ-B had the same items to simplify comparisons of the findings obtained during and after childbirth. Respondents to W-DEQ-B answered the questions by reflecting on the whole process of labour and delivery and the lived experience of childbirth.

The W-DEQ was developed more than 20 years ago, based on data from 196 pregnant women and 166 women after childbirth. Both W-DEQ versions had good reliability and validity during the development process [2]. The internal consistency reliability of the W-DEQ-A administered at 32 weeks of pregnancy was excellent, with Cronbach’s alpha of 0.93. Version B was administered at 2 h and 5 weeks after birth had Cronbach’s alphas of 0.93 and 0.94, respectively. Items that were positively worded (2, 3, 6, 7, 8, 11, 12, 15, 19, 20, 24, 25, 27 and 31) were reversed before individual total scores were calculated.

### The translation and cross-cultural adaptation process

The W-DEQ-A and W-DEQ-B were translated into Kiswahili using translation and cultural adaptation process of Beaton, Bombardier, Guillemin and Ferraz [42], which involves five steps: forward translation, synthesis, back translation, expert committee and pretesting.

#### Forward translation

The English versions of the scale were translated into Kiswahili by the first author.

#### Synthesis

The translated versions were reviewed by a team of six Kiswahili-speaking experts and specialists in reproductive health, midwifery, obstetrics and gynaecology. The team checked for clarity, understandability and wording relative to the Tanzanian context to attain cross-cultural equivalence. The team reached a consensus on each question.

#### Back translation

The forward translation was processed back and forth as experts recommended revisions to the wording of some items. After addressing all these comments, the versions were back-translated into English by two independent bilingual experts. One had a PhD in mental health, and another had expertise in research. The research team members compared the original and back-translated versions for similarities and differences to determine the clarity of the content of the different language versions of the tools. Minor discrepancies were identified, which necessitated some amendments.

#### Expert committee

The tools were e-mailed to 16 Tanzanian native Kiswahili speakers and experts in midwifery, obstetrics and gynaecology, psychiatry, psychology and behavioural science, with more than 5 years of experience in their respective areas of expertise. The experts were invited to rate whether or not each item was essential for measuring FoB. The experts rated each item for necessity on a scale 1–3 (1 ‘not ‘necessary’; 2 ‘useful but not essential’; 3 ‘essential’).

The content validity ratio (CVR) was computed for all individual items. CVR is the proportional level of agreement among experts within a panel rating an item. It is computed as CVR = (Ne – N / 2) / (N / 2), where Ne is the number of subject matter experts indicating an item as essential, and N is the total number of experts. CVR ranges between 1 and -1, and the higher the score, the greater the agreement on the essentiality of an item in a tool. The minimum CVR values to retain items in the tool were determined using the Lawshe Table, which provides critical values based on the number of subject matter experts [43]. For instance, in our study, the number of experts was 16, and if the CVR was more than 0.47, the item was considered to have an acceptable retention value.

Further, the experts were asked to provide recommendations on clarity and understandability for improvement of the questionnaire. A meeting was convened with ten experts who were involved in the rating process to discuss the discrepancies brought up by the experts. One item, which was unclear to the majority of the panellists, was rephrased, and all other uncertainties were clarified. There was agreement among the experts on the cultural relevance of the translated version. Before the tools were piloted, changes made during the meeting were sent for cross-checking and approval to three experts specialized in midwifery, obstetrics, and gynaecology and psychiatry, who participated from the beginning of the validation process.

#### Pretesting

A pilot study was carried out with 31 pregnant and 40 postnatal women attending antenatal and postnatal care in Bagamoyo district health facilities, including the district hospital and two health centres. The pilot study aimed to test the understandability of the questionnaire among pregnant and postnatal women, their interpretation of the items and the rating process using the Likert scale. The research assistants (RAs) read the questions out loud and documented the responses. Rating the items using a 6-point Likert scale was a significant challenge for most women, as the scale had no narratives for each value, just for the two extremities (0 and 5). To address this, a visual scale aid with narratives at points 1–4 was developed to help the women place their responses at the right value depending on their feelings and cognitive appraisal. The Kiswahili items that were unclear to the women were revised without losing the meaning from the original English versions. Together with the newly developed visual scale aid, the tools were re-tested on five pregnant and postnatal mothers in different health facilities. All ambiguities were addressed, a last reformulation of the tools was done, and the final Kiswahili version A and B questionnaires were compiled for data collection.

### Data collection procedure

Six trained RAs collected data through face-to-face interviews using the Kiswahili W-DEQ-Revised tools together with sociodemographic and obstetric questions. The RAs were registered nurse-midwives who were unemployed during data collection. Women were interviewed twice: once during their third trimester, at 32–40 weeks of pregnancy, and again at 4–6 weeks after childbirth. The interviews were carried out in Kiswahili at the selected health facilities. A visual scale was used as an aid for rating the items of W-DEQ-A and W-DEQ-B to avoid discrepancies between different RAs asking questions. The same approach and data collection tools were used at both timepoints.

We trained six RAs in the data collection tools, use of a visual scale, study participant recruitment, and data collection procedures, including ethical principles in data collection. The RAs were registered nurse-midwives who were not employed during data collection. During the actual data collection, RAs recruited study participants, obtained informed consent, and performed data collection through face-to-face interviews in the selected antenatal clinics.

### Data analysis and psychometric evaluation of the Kiswahili W-DEQ tools

Data analysis was conducted using SPSS computer software version 26, with an Analysis of Moment Structure in SPSS version 28 was used for confirmatory factor analysis (CFA). The translated tools were evaluated for both validity and reliability.

#### Sample size assessment, multicollinearity test and multivariate normality test

The sample adequacy was evaluated using the Kaiser Meyer Olkin (KMO) test and the suitability of the data for factor analysis were determined using Bartlett’s test. A sample is adequate and suitable for factor analysis if the KMO value is ≥ 0.60 and Bartlett’s test yields a *p* < 0.01 [44].

Multicollinearity of variables was assessed using the tolerance test, variance inflation factor (VIF) and the condition index. Variables with tolerance values of 0.2, VIF ≤ 5 and condition index values < 15 were maintained for further analysis.

The multivariate normality for versions A and B was measured using Mardia’s multivariate kurtosis [45]. If the Mardia index is less than p(P + 2) (p is the number of observable variables), the sample has multivariate normality [46]. The multivariate outliers were assessed based on their squared Mahalanobis distance in each case. The outliers are the ones whose Mahalanobis d-squared values depart distinctively from others within the dataset [47] and with *p* < 0.001 [48]. The Mardia coefficients for both version A (225.94) and version B (250.91) were lower than p(P + 2) (899). Nevertheless, two multivariate outliers were identified and removed from the analysis.

#### Construct validity

Construct validity was assessed using exploratory factor analysis (EFA) and confirmatory factor analysis (CFA). Before conducting EFA, a CFA using the maximum likelihood estimation method was carried out to test the factorial structure of the original model by Wijma [2] and four other models proposed in previous validation studies for W-DEQ [27, 30, 38, 39]. Several fit of model indices and their criteria in the structural equation models were used to examine the goodness-of-fit index. The chi-squared test with a *p*-value > 0.05 indicates an acceptable fit. Tucker-Lewis Index (TLI) and comparative fit index (CFI) with values above 0.90 represent a good fit. The root means square error of approximation (RMSEA) was also assessed, with values < 0.05 indicating a close fit [49].

After testing the factor structure of the previous studies, an EFA was conducted to extract the factorial structure of the Kiswahili scale using principal component analysis with varimax technique rotation. The criteria for factor retention were: eigenvalues > 1 [50], Catell’s scree plot [51] and factors with item loading above 0.4 [27, 52]. Items with loading below 0.4 were deleted.

Next, CFA was conducted on all items and subscales/constructs created from the EFA to assess whether the data collected confirmed that all items and subscales were indicators of FoB. Modification index fitting was applied to improve structural equation models with inadequate fit. Threshold values higher than 10 points were required to show a significant difference [47]. Within-factor item error terms were correlated after contemplating the theoretical and principal meaning of these modifications.

#### Reliability of translated W-DEQ-A, W-DEQ-B and subscales

Internal consistency of all 33 items and subscales was calculated using Cronbach’s alpha.

A Cronbach’s alpha of > 0.90 is considered excellent, > 0.80 is good, > 0.70 acceptable, > 0.60 questionable and < 0.50 unacceptable [53]. In this study, Cronbach’s alpha above 0.70 was considered a minimum criterion for acceptance.

Moreover, inter-rater reliability was assessed to evaluate the equivalence of different raters scoring participants using the same scale, based on the intra-class correlation coefficient. A coefficient of ≥ 0.7 was considered acceptable inter-rater reliability [54].

#### Subscale analysis

Each emerging subscale extracted from the factor analysis was tested for the total scale score correlation and item score correlation per each item falling in that subscale.

#### Convergent and discriminant validity

The convergent validity of the translated scale was computed using average variance extracted (AVE) and composite reliability (CR) from each factor. A higher value of AVE suggests that factors contribute a lot to the total variance, and an AVE and CR greater than 0.5 and 0.7, respectively, reflect convergent validity [55]. The AVE and CR were calculated using Microsoft Excel.

Discriminant validity was assessed using the Fornell-Larcker criterion (1981) by comparing the square root of each AVE and the correlation values of the factors. A square root of AVE higher than the correlation coefficient between the factors suggests discriminant validity [56, 57].

## Results

### Sociodemographic and obstetric characteristics

Of 694 eligible pregnant women who agreed to participate, 625 were followed up in a second interview. The response rates were 98.9% and 90.1% for pregnant and postnatal women, respectively. The median age was 26 years (interquartile range = 11; range 14–46). Most women (81.6%) had primary education and were employed (70.6%) and married (73.2%). A large proportion (67.9%) had planned pregnancies. Half of them had given birth more than once before and 61.3% had never experienced any childbirth complications.

### Content and face validity

The CVR was calculated for each item. Items with CVR less than 0.47 were identified as non-essential. This cut-off point was based on the total number of panellists (*N* = 16). One item out of 33 was marked as non-essential. The item was not removed, though it is recommended that non-essential items are deleted. The item was retained and modified based on the opinion of subject matter experts in the first round. The item was ‘In general, I will be relaxed during labour and delivery’. The concept of ‘being relaxed during labour’ was not translated well into Kiswahili; hence it was difficult for the experts to contextualize its meaning. During the consensus meeting, a more appropriate wording was proposed. For each questionnaire version, thirteen items had a CVR of 1.00, thirteen a score of 0.87, six a score of 0.73, and one a score of 0.33.

During the piloting of the W-DEQ-A and W-DEQ-B, the concept of labour and delivery being ‘fantastic, funny, and a surrender of control of the body’ was not clear to the women during the face-to-face interviews. This could be due to translation problems or incorrect wording of the question. A rewording of the items was performed and re-tested on pregnant and postnatal women for clarity and understandability.

### Construct validity

#### Confirmatory factor analysis for the previous studies

Table 1 shows that neither the one-factor model proposed by Wijma nor the three-, four- and six-factor models presented in previous studies had satisfactory fit indices for our sample for either W-DEQ-A or W-DEQ-B. Consequently, we decided to carry out an EFA on our dataset to reveal the factor structure, followed by a CFA to assess the model fit between latent factors and observed variables.

**Table 1:** Confirmatory factor analysis grounded on structures from other countries

Factor	Country of study	Number of items	*X*^*2*^*/df*	*P-value*	RMSEA	CFI	TLI
**W-DEQ-A**							
One factor (original scale)	Sweden [2]	33	4.04	< .001	.066	.64	.62
Three factors	Malawi [38]	26	2.81	< .001	.051	.81	.83
Four factors	Australia [27]	32	3.189	< .001	.056	.75	.73
Five factors	Kenya [39]	24	3.38	< .001	.059	.82	.79
Six factors	Iran [30]	32	2.41	< .001	.045	.84	.83
**W-DEQ-B**							
One factor (original scale)	Sweden [2]	33	4.86	< .001	.079	.59	.57
Three factors	Malawi [38]	26	3.19	< .001	.059	.81	.79
Four factors	Australia [27]	32	3.58	< .001	.06	.74	.72
Five factors	Kenya [39]	24	3.98	< .001	.07	.79	.77
Six factors	Iran [30]	32	2.97	< .001	.056	.81	.79

*CFI* Comparative fit index, *df* Degrees of freedom, *RMSEA* Root mean square error approximation, *TLI* Tucker-Lewis index; *X*^*2*^ Chi-squared

#### Exploratory factor analysis

An EFA was conducted for 33 items in the W-DEQ-A. The KMO value for the sample adequacy was 0.875, and Bartlett’s test of sphericity was significant (*p* < 0.001), indicating an adequate sample for the EFA. The EFA identified seven factors with eigenvalues > 1 and factor loading above 0.4. Twenty-nine (29) items loaded to seven factors, and four items did not load to any factors: longing for a child, surrender control of the body, funny and self-evident. The seven factors explained almost 50% of the total variance in the dataset. They were named based on the conceptual contents of the loaded items: fear (5 items), lack of self-efficacy (6), lack of positive anticipation (7), isolation (3), concerns for the baby/riskiness (2), negative emotions (3), and lack of positive behaviours (3). Among the seven factors, factor 5 (concerns for the baby) had less than three items loading with > 0.4. The factor loadings ranged from 0.427 to 0.856. Table 2 presents the details of the EFA.

**Table 2:** W-DEQ-A, seven factors and factor loading, eigenvalues (*n* = 694)

Item	Fear	Lack of self-efficacy	Lack of positive anticipation	Isolation	Concerns for the baby/Riskiness	Negative emotions	Lack of positive behaviours
6. Afraid	**0.712**	0.212	0.052	0.085	0.080	0.030	0.108
12. Tense	**0.684**	0.117	0.083	0.109	0.121	0.075	0.060
19. Panic	**0.684**	0.100	0.126	0.078	0.080	0.207	0.034
25. Behave badly	**0.588**	0.013	0.130	0.013	0.039	0.103	0.272
8. Weak	**0.449**	0.119	0.092	0.254	0.067	0.182	0.120
5. Confident	0.111	**0.730**	0.113	0.115	0.080	0.065	0.012
22. Self-confidence	0.134	**0.635**	0.146	0.120	0.071	0.125	0.234
23. Trust	0.024	**0.627**	0.213	0.043	-0.040	0.065	0.379
4. Strong	0.147	**0.619**	0.106	0.111	0.060	-0.003	-0.215
9. Safe	-0.136	**0.461**	0.262	0.455	0.008	0.096	-0.030
29. Natural	0.302	**0.442**	0.104	0.253	-0.025	-0.133	-0.003
14. Proud	0.061	0.005	**0.743**	0.164	0.014	0.034	0.037
18. Happy	0.103	0.132	**0.701**	-0.132	0.100	0.101	0.112
13. Glad	0.099	0.134	**0.648**	0.048	-0.059	0.157	0.022
16. Composed	0.223	0.286	**0.523**	0.058	0.068	-0.083	0.129
10. Independent	-0.049	0.340	**0.487**	0.377	-0.011	-0.056	0.040
17. Relaxed	0.097	0.393	**0.423**	-0.148	0.042	0.000	0.234
1. Fantastic	0.094	0.215	**0.405**	-0.031	0.141	0.376	-0.232
11. Desolate	0.343	0.038	0.067	**0.598**	0.062	-0.173	-0.027
7. Deserted	0.149	0.026	-0.037	**0.583**	0.066	0.303	0.091
15. Abandoned	0.357	-0.049	-0.013	**0.426**	0.007	0.148	0.224
30. Self-evident	0.274	0.166	0.001	**-0.180**	-0.079	0.296	0.178
33. Child will be injured	0.136	0.094	0.054	0.078	**0.836**	0.054	0.035
32. Child will die	0.140	0.033	0.039	0.021	**0.829**	0.006	0.133
24. Painful	0.067	0.049	0.067	0.025	-0.049	**0.673**	0.168
2. Frightening	0.353	0.024	0.026	0.118	0.110	**0.489**	0.004
3. Lonely	0.427	0.007	0.042	0.128	0.127	**0.485**	0.041
28. Funny	0.049	0.217	0.147	0.101	0.058	0.159	0.146
20. Hopelessness	-0.046	0.122	0.080	0.106	0.182	0.153	**0.577**
27. Lose control	0.365	0.006	0.161	0.098	0.004	-0.137	**0.575**
31. Dangerous	0.302	0.115	0.032	0.357	0.056	-0.212	**0.420**
26. Surrender control of the body	-0.090	-0.092	-0.141	-0.165	-0.187	-0.007	-0.080
**Eigenvalues**	**6.1**	**2.6**	**1.4**	**1.3**	**1.2**	**1.1**	**1.1**
**Variance %**	**20.2**	**7.9**	**5.7**	**4.8**	**3.9**	**3.7**	**3.6**

#### Confirmatory factor analysis for the current study

Since the number of items and contents of W-DEQ-A and W-DEQ-B are similar, EFA was carried out on W-DEQ-A to establish its factorial structure. Then, a CFA of W-DEQ-B was performed to explore if the factorial structure produced by the EFA had a good fit for W-DEQ-B. The seven-factor model showed a good fit with one index (X^2^/df), marginal fit with three indices (RMSEA, CFI and TLI) and poor fit with one index with a *p* value less than 0.005 (Model 1). The model was modified. We covaried two correlated-error terms (e19–e20) with higher modification indices and contextual similarity between items to produce Model 2. However, the modified model did not improve the goodness-of-fit indices much. Then, two other correlated-error terms (e9–e10) were covaried to yield Model 3. The model showed better fit indices but was still marginally lower than recommended criteria for acceptable model fit. Lastly, based on the modification indices, we added three error covariances terms (e19–e20, e9–e10, e17–e18) to generate Model 4, which showed acceptable fit indices except for a *p*-value < 0.05 and TLI < 0.90. Detailed results are provided in Table 3.

**Table 3:** Goodness-of-fit indices for four models of the W-DEQ-B (*n* = 625)

Factor model	X^2^	df	X^2^/df	*p-*value	RMSEA	CFI	TLI
Model 1: Seven-factor model (theoretical model)	914	356	2.57	< .001	.050	.87	.85
Model 2: Modified seven-factor model with one error covariance terms (e19–e20)	856	355	2.41	< .001	.048	.88	.86
Model 3: Modified seven-factor model with two error covariances terms (e19–e20, e9–e10)	825	354	2.33	< .001	.046	.87	.89
Model 4: Modified seven-factor model with three error covariances terms (e19–e20, e9–e10, e17–e18)	797	353	2.26	< .001	.045	.90	.88

*CFI* Comparative fit index, *df* Degrees of freedom, *RMSEA* Root mean square error approximation, *TLI* Tucker-Lewis index, *X*^*2*^ = chi-squared

**Fig. 1:**
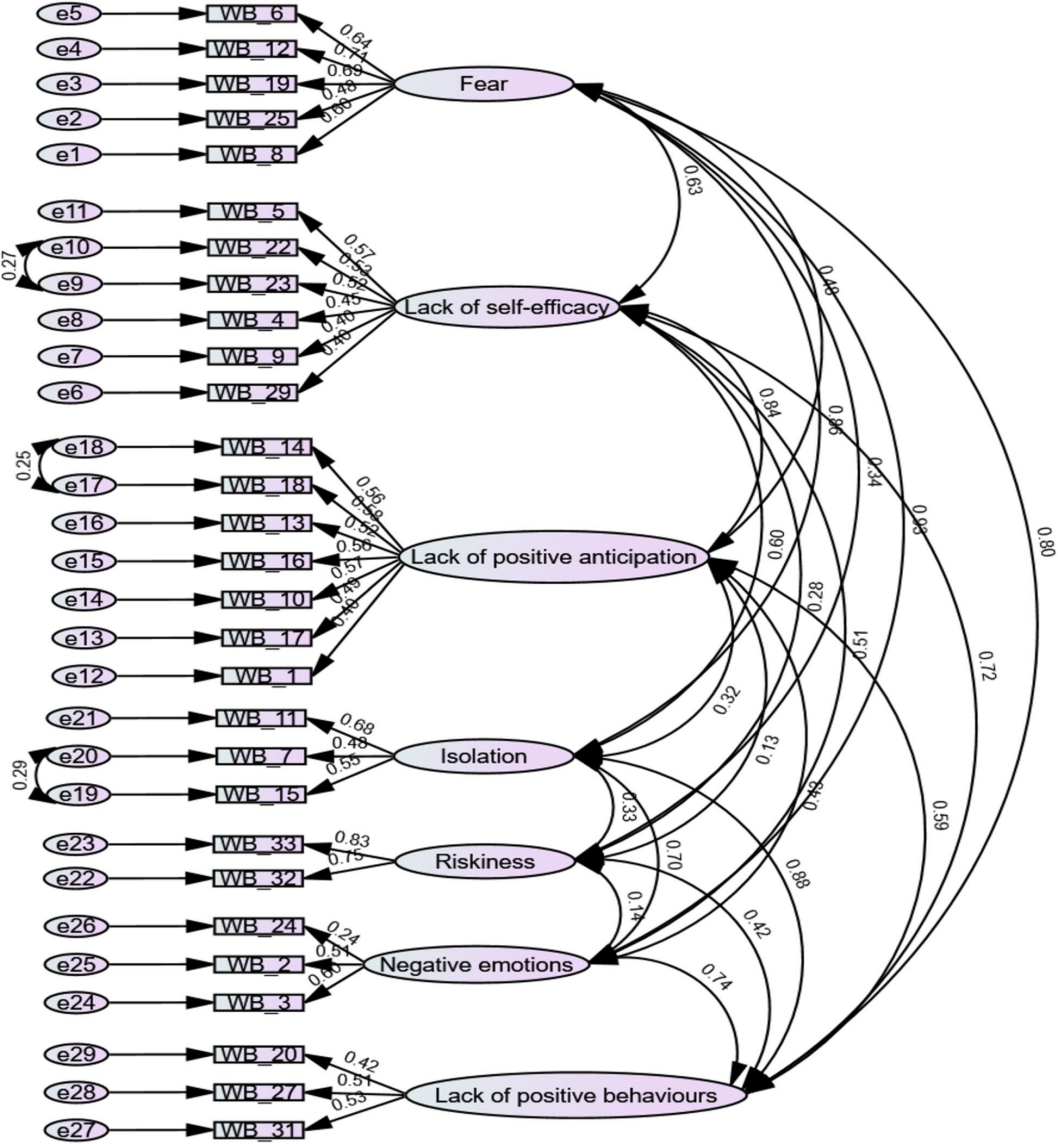
Confirmatory factor analysis path diagram for W-DEQ-B (7-factor model, 29 items)

Therefore, the seven-factor model with 29 items and three error covariance terms (Model 4) was considered acceptable for further discussion while maintaining a high number of items from the original scale. See further in Fig. 1.

### Reliability of translated W-DEQ A and W-DEQ-B

The Cronbach’s alpha was 0.832 and 0.849 for the 33 translated W-DEQ-A and W-DEQ-B items, respectively. The internal consistency reliability improved slightly for both W-DEQ-A (α = 0.850) and W-DEQ-B (α = 0.867) with 29 items.

Regarding the internal consistency of the items included in the subscales of both versions, the results indicated that two factors – fear and lack of positive anticipation – had good Cronbach’s alphas (> 0.70) for both W-DEQ-A and W-DEQ-B, while lack of self-efficacy, concern about the baby/riskiness and isolation had moderate values and lack of positive behaviours had low values of Cronbach’s alpha. See Table 4 for more details.

**Table 4:** Reliability estimates of Cronbach’s alpha for factors and items

		**W-DEQ-A**	**W-DEQ-B**
**Factors**	**Items**	**Cronbach’s alpha**	**Cronbach’s alpha**
W-DEQ total	33	.832	.849
Fear	5	.740	.750
Lack of self-efficacy	6	.700	.660
Lack of positive anticipation	7	.730	.720
Isolation	3	.520	.660
Concerns for the baby/Riskiness	2	.660	.760
Negative emotions	3	.480	.420
Lack of positive behaviour	3	.450	.470
Whole scale	29	.850	.870

Further, the intra-class correlation coefficient was 0.98 for versions A and B, indicating inter-rater reliability.

### Subscale correlation

The results indicated that the total scores on the W-DEQ-A and W-DEQ-B were strongly correlated with fear (*r* = 0.787, *p* < 0.001) and weakly correlated with concerns for the baby (*r* = 0.40, *p* < 0.001). Lack of self-efficacy was moderately correlated with a lack of positive anticipation (*r* = 0.55, *p* < 0.001), fear was moderately correlated with isolation (*r* = 0.48, p < 0.001), negative emotions (*r* = 0.48, *p* < 0.001) and lack of positive behaviours (*r* = 0.43, *p* < 0.001). All the remaining factors were weakly correlated with each other.

#### Convergent and discriminant validity

As shown in Table 5, the AVE values for six factors out of seven in both versions were below the proposed cut-off. This indicates that convergence was not met by most of the factors. Only the concerns for the baby factor showed acceptable convergent validity with AVE and CR greater than 0.5 and 0.7, respectively. Fear and lack of positive anticipation factors had acceptable CRs, greater than 0.7, but did not meet the minimum required AVE to suggest convergent validity.

**Table 5:** The average variance extracted (AVE), composite reliability (CR), square root of AVE and matrix of correlations between factors for W-DEQ-A and W-DEQ-B

Factors	Items	AVE	CR	A	B	C	D	E	F	G
**W-DEQ- A**										
Fear (A)	5	.38	.75	**.61**						
Lack of self-efficacy (B)	6	.28	.86	.35	**.53**					
Lack of positive anticipation (C)	7	.29	.98	.35	.55	**.54**				
Isolation (D)	3	.27	.53	.48	.24	.19	**.52**			
Concerns for the baby/Riskiness (E)	2	.51	.72	.29	.18	.17	.17	**.71**		
Negative emotions (F)	3	.26	.50	.48	.20	.21	.33	.18	**.51**	
Lack of positive behaviour (G)	3	.25	.48	.43	.32	.29	.35	.24	.29	**.50**
**W-DEQ-B**										
Fear (A)	5	.40	.76	**.63**						
Lack of self-efficacy (B)	6	.23	.64	.43	**.48**					
Lack of positive anticipation (C)	7	.28	.73	.36	.47	**.53**				
Isolation (D)	3	.33	.59	.56	.34	.19	**.58**			
Concerns for the baby/Riskiness (E	2	.63	.77	.26	.22	.09	.22	**.80**		
Negative emotions (F)	3	.23	.48	.58	.29	.29	.35	.08	.**48**	
Lack of positive behaviour (G)	3	.28	.73	.50	.41	.33	.47	.26	.34	**.49**

Values in bold are the square root of AVE

*AVE* Average variance extracted, *CR* Composite reliability

The square root of AVE of all factors in both versions was higher than the correlation between the factors, suggesting discriminant validity.

## Discussion

This study aimed to evaluate the reliability and validity of the Kiswahili versions of W-DEQ-A and W-DEQ-B in the Tanzanian context. The results revealed the Kiswahili versions to be reliable tools with good internal consistency and inter-rater reliability in measuring FoB before and after childbirth in Tanzanian women. The results are consistent with several other studies with acceptable Cronbach’s alphas [24–26, 30–34, 36].

In our study, four items – ‘longing for a child’, ‘funny’, ‘self-evident,", and ‘surrender control of the body’ –as they did not load to any factor. This indicates that these items are not measuring the same construct of fear as others. This was in line with other studies where more or less the same items loaded less than the minimum acceptable coefficient criterion, leading to their deletion. In two other studies done in the United Kingdom and Italy, ‘funny’, ‘surrender control of the body’, and ‘"self-evident were excluded from the scale [28, 32]. ‘Longing for a child’ was distinct in this study, with the lowest overall mean score and coefficient, indicating that most women are enthusiastic about having a baby. However, the items were not deleted in other studies, as they correlated with other items [24, 25, 27, 37]. The discrepancy could be due to the challenges in translation from English to Kiswahili, where it was difficult to find the appropriate Kiswahili word, leading to difficulty in understanding these items. A further explanation could be the way childbirth is conceptualized in the study context. For instance, in Tanzania, labour and childbirth being ‘funny" was not well conceptualized by women. The item ‘funny"’ has been modified to fit the context of other studies [26, 27]. From the findings of this study, contextualizing the questionnaire is advocated to ensure consistency and true value of the findings in measuring FoB.

In the current study, the findings suggested multidimensionality of the W-DEQ-A and W-DEQ-B with seven factors. The first factor referred to feelings of fear, comprising items such as being perceived as weak, feeling tense or feeling panic. Fear also appeared to be related to the feeling of behaving badly during childbirth. The label ‘fear’ was consistent with previous findings [24, 30–33]. The second factor was a lack of self-efficacy. The factor consisted of items that indicated how feeling strong, safe, confident, having self-confidence, trust in oneself and others along the course of childbirth, and perceiving childbirth as a natural process were related to the women’s expectations and experiences of childbirth. The factor label was similar to those used in other studies [24, 30]. The third factor, lack of positive anticipation, focused on expectations and experiences of childbirth as an exciting event, with emotions ranging from being happy, glad, composed, independent, feeling relaxed during the process, being proud of oneself, and perceiving childbirth as a fantastic life event. The label was in line with those used in previous studies [24, 30, 33]. The fourth factor referred to the expectation and experience of isolation, composed of the items indicating a sense of being desolate, deserted, and abandoned, either by health care providers or by a partner or significant other. The factor labelling was similar to that in other studies [25, 33]. Factor five focused on concerns for the baby; that the baby might be injured or die during childbirth. It indicates that expectations of childbirth are linked to the thoughts that something might happen to the baby. The factor label was based on previous studies [24, 30, 31, 33, 37]. The sixth factor was about having negative emotions or feelings about childbirth. The items in the factor described how the sensation of physical pain, perceiving childbirth as frightening or having feelings of loneliness could relate to women’s expectations and childbirth experiences. The label given to the factor was coherent with the study done by Pallant and colleagues [25]. The final factor identified was a lack of positive behaviour. Women perceived childbirth as a dangerous life event, losing control and hope during the childbirth process. The label for the factor was guided by a previous study [37]. The number of factors found in the current study (seven) is unusual, with other studies finding three [32], four [25, 33, 34], five [35], six [24, 26–28, 30] and nine [31].

When the relationship patterns were compared to assess the convergent validity of the Kiswahili translated W-DEQ-A and W-DEQ-B, we found that the concern for the baby factor had adequate convergent validity. This indicates that the feeling that the baby would be injured and the feeling that it would die had positive interrelationships with each other. On the other hand, the analysis revealed insufficient support for convergent validity of the other six factors, meaning that items loading to different factors did not correlate with each other within their latent variable/parent factor. The latent variables are not well-explained by the observed items. This was also revealed when the correlation of subscales was analysed. There was a poor correlation between subscales. The findings are contrary to the results of a study done in Japan, where the Japanese W-DEQ version showed overall convergence with other tools [34]. The difference could be how participants perceive pregnancy, labour and childbirth. For instance, items like feeling happy, glad, proud or composed might be contextualized differently, affecting correlation. Also, lack of consistency in responding between one item and another might have brought about the discrepancy. In addition, being a homogenous population with almost the same education level, occupation, religion and other shared cultural aspects might have contributed to the difference compared with other studies. Regarding discriminant validity, the findings revealed that factors in the scales shared more variance with their latent variables than with other constructs outside their parent factor [57].

To find the best items for measuring FoB in Tanzanian women, four out of 33 items were removed from the scale. The acceptable fit model encompasses 29 items. The *p*-value in our structural equation model did not fit the model well. However, most indices were within the acceptable fit, ensuring the tool had construct validity. The number of items retained was consistent with other international studies that deleted misfitting items. However, the number of items removed from previous studies differed from this study. The study done in Iran, 32 items were suggested to measure FoB, with one item removed to find the best fit model [30]. In another study done in Norway, 8 out of 33 items were removed from the scale to get an acceptable fit model of 25 items [24]. A study in Italy removed even more items from the mode, yielding 14 items in the final structure with adequate psychometric properties [32].

The factors had differing correlations. However, the fear factor had an acceptable Cronbach’s alpha and strongly correlated with the total score, indicating that the factor could be closer to measuring FoB than the entire W-DEQ scale.

### Strengths and limitations of the study

The major strength of the study was the rigorous process undertaken in the translation and validation of the questionnaires. Also, using a visual aid with more narratives along the scale improved ease of understanding of the Likert scale in the W-DEQ tools. Further, the sample was large enough for the validation of the tools. Collecting data during pregnancy and following up after childbirth gave a chance to identify factorial structure in both versions of W-DEQ. Another strength is that the Kiswahili W-DEQ-Revised was tested among women who anticipated a vaginal delivery and had no history of caesarean sections. To our knowledge, this is the first study to evaluate the reliability and validity of Kiswahili W-DEQ tools for measuring FoB before and after childbirth in Tanzania. A limitation was the use of non-probability sampling, where the sample may not be representative of pregnant women and postnatal mothers. Also, test–retest reliability was not assessed, as we did not manage to interview women twice during pregnancy or twice after childbirth. We interviewed them during different periods, i.e., during pregnancy and after childbirth. Therefore, test–retest reliability could not be measured.

## Conclusion

The Kiswahili W-DEQ version A and B-Revised is a reliable and multidimensional tool with seven subscales. Twenty-nine items with good psychometric properties were identified to best measure FoB among Tanzanian women before and after childbirth. This revised Tanzanian W-DEQ may provide researchers with a more refined and psychometrically sound questionnaire to assess the FoB construct in pregnant and postnatal women. Further research is recommended to test the tool on a heterogeneous population and pay special attention to convergent validity, as there was some inconsistency in our findings. Also, we recommend further studies to validate the scale for all pregnant women irrespective of gestational age since the W-DEQ-A was validated in pregnant women in the third trimester of pregnancy only.

## Data Availability

The datasets supporting the conclusions of the article can be available on a reasonable request to the first author and the chairperson of the senate, research, and publication committee of Muhimbili University of Health and Allied Sciences (MUHAS) with this email: drp@muhas.ac.tz.

## Abbreviations

AVE: Average extracted variance
CFA: Confirmatory factor analysis
CFI: Comparative fit index
CR: Composite reliability
CVR: Content validity ratio
EFA: Exploratory factor analysis
FoB: Fear of childbirth
RA: Research assistant
RMSEA: Root mean square error approximation
TLI: Tucker-Lewis Index
W-DEQ-A/B: Wijma Expectancy/Experience Delivery Questionnaire versions A and B
